# A Comparison of Three Techniques in Neonatal Circumcision: Artery Forceps, Bone-cutter, and Gomco Clamp Methods

**DOI:** 10.4314/ejhs.v35i1.5

**Published:** 2025-01

**Authors:** Victor Ifeanyichukwu Modekwe, Chukwubunna Ezeifedikwa, Evan Therese Nwosu, Ezekiel Uchechukwu Nwankwo, Okechukwu Hyginus Ekwunife, Jideofor Okechukwu Ugwu, Charles Chidiebele Maduba, Ugochukwu Uzodimma Nnadozie, Ugochukwu Stanley Ezidiegwu, Chuka Abunike Ugwunne

**Affiliations:** 1 Immaculate Heart of Mary Specialist Hospital Nkpor Anambra, State; 2 Department of Surgery, Nnamdi Azikiwe University, Awka; 3 Department of Surgery, Nnamdi Azikiwe University Teaching Hospital, Nnewi; 4 Department of Surgery, University of Nigeria Teaching Hospital, Enugu; 5 Department of Surgery, Alex Ekwueme Federal University Teaching Hospital, Abakaliki

**Keywords:** Gomco, artery-forceps, guillotine, bone-cutter, Paediatric penile

## Abstract

**Background:**

Neonatal circumcision is the oldest and most common surgical procedure The safety, ease, and outcomes of various methods of surgical procedures for neonatal circumcision have become increasingly the focus in the reviews of this procedure. This study aimed to identify the easy, safe and acceptable method for neonatal circumcision.

**Methods:**

This prospective study involved 357 male neonates, divided into three groups based on the methods used for neonatal circumcision: artery-forceps, bone-cutter, and Gomco methods. Clamps were uniformly applied for 7 minutes (420 seconds). The study assessed procedure time, primary and reactionary bleeding, and outcomes using the adapted Paediatric Penile Perception (PPP) score. Data were analyzed using SPSS version 23, with a p-value set at <0.05.

**Results:**

Each group consisted of 119 neonates. The bone-cutter method was the fastest (590.2 ± 60.14 seconds), while the Gomco method was the slowest (624.2 ± 55.16 seconds, p<0.001). Primary bleeding occurred most frequently with the artery-forceps method (37 out of 119), and least with the bone-cutter (p<0.001). Only the artery-forceps group had reactionary bleeding (p=0.018). The bone-cutter and Gomco methods had the highest PPP mean scores: 11.91 ± 0.390 and 11.87 ± 0.566, respectively (p<0.001).

**Conclusion:**

The bone-cutter method is the fastest, safest, and produces the best cosmetic outcomes of the three methods studied.

## Introduction

Circumcision is the surgical removal of the prepuce (1), and it is the oldest and most common surgical procedure (2). Typically, it is performed in the neonatal period (2-4). Medical indications for circumcision include paraphimosis, phimosis, balanoposthitis, and recurrent urinary tract infections (UTIs). However, the most common indications are socio-cultural and religious (3, 4).

There are potential complications associated with circumcision, such as penile amputation, urethrocutaneous fistula (UCF), glansular injury, meatal injury, hemorrhage, ring retention and migration, iatrogenic scrotal skin migration onto the penile shaft, phimosis, buried penis, and glansular necrosis (5,6). Despite the ongoing debates for and against circumcision, the procedure continues to be widely performed (3, 4).

For pediatric surgeons, circumcision is an important procedure, especially given the high complication rate resulting from those performed by “untrained” practitioners. Such cases typically require more technically demanding procedures to correct the ensuing complications.

Neonatal circumcision is increasingly being performed under anesthesia or operative analgesia, such as EMLA® cream, dorsal penile nerve block, penile ring block, or oral ketamine (2,7). Several methods have been developed for the procedure, including the dorsal slit, excision (with various modifications, such as sleeve resection), Guillotine, forceps-guided, and clamp methods (8). Each method has its variations, modifications, and associated risks (8,9, 10), and any of the complications mentioned above can occur with them.

The Guillotine method, commonly used by traditional circumcisers, lacks protection for the glans and does not include an in-built hemostatic mechanism, leading to a higher risk of blood loss and injury from suture ligations of bleeding vessels (8). The forceps-guided method often uses a bone-cutter. This method provides some protection to the glans and has an in-built hemostatic mechanism, but its safety depends on the skill of the practitioner. The bone-cutter shields the glans before trimming the excess prepuce (8). One of the advantages of this method is that it does not leave a ring, serving as a “one-stop” procedure with a built-in hemostatic mechanism to protect the glans.

Occlusive methods (such as the Plastibell and Gomco clamp) provide high protection for the glans when the prepuce is crushed and excised. The Plastibell is the most commonly used in our region, but it has complications, including ring retention and proximal migration. These issues often arise from improper sizing of the Plastibell, typically estimated by visual inspection (11). Furthermore, mothers often express concerns about their babies going home with a ring still present over the glans. In some cases, these rings require later removal in the hospital. The Gomco clamp, another occlusive method, does not leave a ring after the procedure, which is an attractive feature (8,9, 10).

Maternal preferences regarding circumcision methods and outcomes have become an important factor in decision-making (12). There is growing interest in methods that do not require neonates to go home with a ring that will eventually fall off (13). At our center, we recently introduced the Gomco clamp and forceps-guided methods (bone-cutter and artery forceps). We modified all three methods by keeping the crushing mechanism in place for seven minutes, equivalent to the average bleeding time, to improve hemostasis and reduce the need for post-procedure bleeding control. These three methods offer the advantage of being “one-stop” procedures without a ring that will fall off later.

The Gomco clamp protects the glans with a metallic bell while the plate tightens to strangulate the prepuce, facilitating safe excision (8, 10). The Gomco clamp has been shown to be as effective, if not superior, to the Plastibell® technique, which is more common in our region (13). It is the most widely used method in the United States, where the complication rate is 0.2% (14). The forceps-guided methods—either with bone-cutter or artery forceps—shield the glans on one surface while the prepuce is excised flush on the other side. The bone-cutter has a groove that protects the glans (8). The artery forceps method, recently introduced at our center, uses mechanical pressure to shift the glans away from the clamping edges (9, 10). Both methods have technical challenges but generally lead to favorable outcomes.

This study aimed to compare the three techniques to evaluate their relative safety, ease of use, and which method yields the most desirable outcomes. Specifically, it focused on primary and reactionary hemorrhage rates, operation times, and maternal preference regarding the aesthetic appearance of the circumcised penis.

## Patients and Methods

This was a randomized controlled trial conducted at Immaculate Heart of Mary Specialist Hospital (IHMSH), Nkpor, Anambra State, Nigeria, from April 2021 to July 2022 (16 months). IHMSH is a mission hospital that provides primary, secondary, and some tertiary health care.

The study involved male neonates (<28 days old) undergoing circumcision, with the consent of their mothers or caregivers. Inclusion criteria were male neonates born at full term. Exclusion criteria included preterm neonates, those with congenital penile anomalies, penile rashes, jaundice, or a history of bleeding disorders, as well as neonates whose caregivers refused consent. Using an online sample size calculator, the required sample size for each group was at least 114 neonates. Ethical approval was obtained from the Health Research and Ethics Committee of Nnamdi Azikiwe University Teaching Hospital (NAUTH/CS/66/VOL.14/VER.3/146/2021/033).

Informed consent was obtained from each mother or caregiver. The study, including the types of procedures and associated risks, was explained to them, and their concerns were addressed. The mothers were informed that the circumcision method would be chosen randomly. Randomization was performed using a convenient sampling method. Ballots labeled A, B, and C were placed in a bag, with the first neonate assigned to the first ballot, the second to the second, and so on. Ballot A designated the artery forceps group, Ballot B the bone-cutter group, and Ballot C the Gomco clamp group. The mothers were blinded to the assigned method.

Procedure was carried out under peile ring block. Preputial transillumination (14) was used to improve the safety of the procedure in addition to palpation. The outcome measures were time of procedure, bleeding and penile appearance using a modification of Paediatric Penile (PPP) score (15). The outcome measures were time to complete the procedure, primary and reactionary bleeding, and cosmetic appearance. A modification of the Pediatric Penile Perception (PPP) score was used to assess early post-procedure appearance after one week (16).

## Results

A total of 357 neonates were recruited, with 119 in each group. The mean age of the neonates was 12.6 (+4.4) days, and the mean weight was 3.7 (+0.57) kg (Table 1). The bone-cutter group had the shortest procedure time (590.2 ± 60.14 seconds), which was statistically significant (<0.001).

**Table 1:** Comparison of Mean age and weight of subjects

Parameters	Artery Forceps group	Bone-Cutter group	Gomco Clamp group	TOTAL	P value
Number of Subjects per group	N = 119	N = 119	N = 119	N = 357	-
Mean Age (days)	12.9 ±4.11	12.4 ±3.98	12.5 ±5.06	12.6 ±4.4	0.693
Mean Weight (Kg)	3.6 ± 0.55	3.8 ±0.57	3.7 ±0.59	3.7 ±0.57	0.140
Duration of Procedure (Seconds)	596.0 ±58.5	590.2 ±60.14	624.2 ±55.16	603.4 ±59.67	<0.001

There were 56 cases of breakthrough primary hemorrhage: 37 in the artery forceps group, 14 in the Gomco clamp group, and 5 in the bone-cutter group. The difference was statistically significant. Four cases of reactionary hemorrhage occurred in the artery forceps group. There was one incident of glansular injury in the artery forceps group (see Table 2).

**Table 2:** Bleeding complications among groups (1 case of glansular injury for artery forceps)

Type of Bleeding Complications		Circumcision Methods			TOTAL	P-Value

		Artery Forceps	Bone-cutter	Gomco Clamp		
Breakthrough / Primary Bleeding	YES	37	5	14	56	
	NO	82	114	105	301	<0.001
TOTAL		119	119	119	357	
Reactionary Bleeding	YES	4	0	0	4	
	NO	115	119	119	353	.018
TOTAL		119	119	119	357	

In controlling primary hemorrhage, ligation was the most effective method in about half of the cases, followed by pressure. Adrenaline solution was more effective in the Gomco clamp group compared to the artery forceps and bone-cutter groups, with significant differences noted (see Table 3).

**Table 3:** Control of bleeding across groups

		Circumcision Method				

Control of Bleeding	Methods	Artery Forceps	Bone-Cutter	Gomco Clamp	TOTAL	P-Value
	Not applicable	82	114	105	301	
	Pressure	15	2	1	18	
Breakthrough / Primary	Adrenaline solution	6	1	5	12	
Bleeding	Ligation	16	2	8	26	<.001
TOTAL		119	119	119	357	
	Not applicable	115	119	119	353	
	Pressure	1	0	0	1	
Reactionary Bleeding	Adrenaline Solution	3	0	0	3	.088
	Ligation	0	0	0	0	
TOTAL		119	119	119	357	

The modified PPP scores were consistently higher for the Bone-cutter group in the perception of the mothers and the investigator. It is lowest with the Artery Forceps group at all the parameters (see Table 4 & 5).

**Table 4:** Count of Paediatric penile perception (PPP) from maternal and Investigator perspective on 7th day post-procedure

Penile Features		Very satisfied (3)		Satisfied (2)		Dissatisfied (1)		Very Dissatisfied (0)		¥	P-value
		Mat	Inv	Mat	Inv	Mat	Inv	Mat	Inv		
Meatal shape and position	Artery forceps	104	111	14	7	1	1	0	0	.171	<.001
	Bone cutter	118	119	1	0	0	0	0	0		
	Gomco	117	118	2	1	0	0	0	0	(.153)	(.004)
Shape of the glans	Artery forceps	104	106	14	12	1	1	0	0	.175	<.001
	Bone cutter	116	117	3	2	0	0	0	0		
	Gomco	116	117	3	2	0	0	0	0	(.177)	(<.001)
Shape of the penis	Artery forceps	105	101	12	16	2	2	0	0	.147	.005
	Bone cutter	116	115	3	4	0	0	0	0		
	Gomco	115	115	4	4	0	0	0	0	(.186)	(<.001)
General cosmetic appearance	Artery forceps	93	88	24	27	2	3	0	1	.211	<.001
	Bone cutter	114	114	5	5	0	0	0	0		
	Gomco	112	113	6	5	1	1	0	0	(.266)	(<.001)

Mat – maternal; Inv – investigator; ¥ - Spearman ranking value; (…) – for investigator PPP test values.

**Table 5:** Comparison of Mean Total Paediatric Penile Perception (PPP) scores for the three groups for the Maternal and the Investigator

Scores	Artery Forceps	Bone Cutter	Gomco	ANOVA	P-value
Mean Maternal total PPP scores	11.39 (SD 1.136)	11.91 (SD .390)	11.87 (SD.566)	16.963	<.001
Mean Investigator total PPP scores	11.42 (SD 1.204)	11.92 (SD .381)	11.89 (SD.517)	14.951	<.001

## Discussion

The neonates were equally distributed across the three groups with similar age and weight. The procedure time was fastest in the bone-cutter group, consistent with findings from previous studies (13). The bone-cutter method also showed the lowest incidence of bleeding complications, while the artery forceps group had the highest. Notably, reactionary bleeding was absent in both the bone-cutter and Gomco clamp groups, making them safer options for neonatal circumcision. Bleeding post-circumcision has led to morbidity and mortality if not managed well (17,18).

The bone-cutter method also had the best cosmetic outcome, as assessed by the PPP scores, followed by the Gomco clamp group. The artery forceps group had the lowest satisfaction rate.

Overall, the bone-cutter method proved to be the most efficient and safest option with the least bleeding complications and best aesthetic results. The Gomco clamp method is also a safe and effective option, while the artery forceps method should be reserved for skilled practitioners due to its higher risk of complications. This is similar to the studies of Talabi et al (13) and Makhlouf GA et al (19). Thus, we recommend the bone-cutter method for neonatal circumcision, followed by the Gomco clamp. The artery forceps method should be used by experienced hands with careful attention to hemostasis.

## Figures and Tables

**Figure 1:**
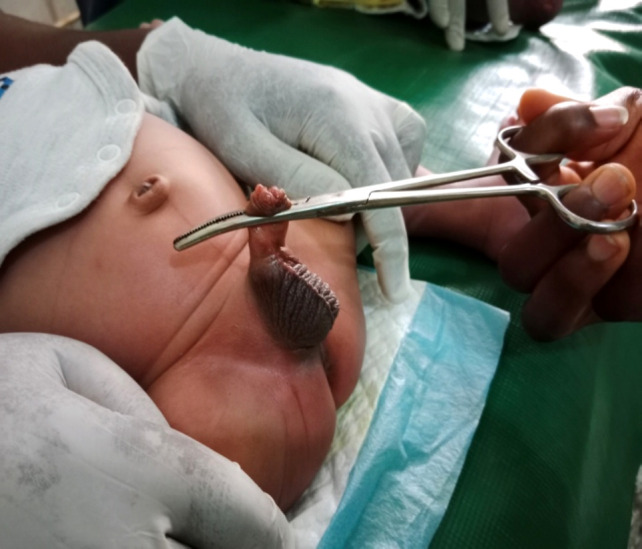
Artery Forceps circumcision

**Figure 2:**
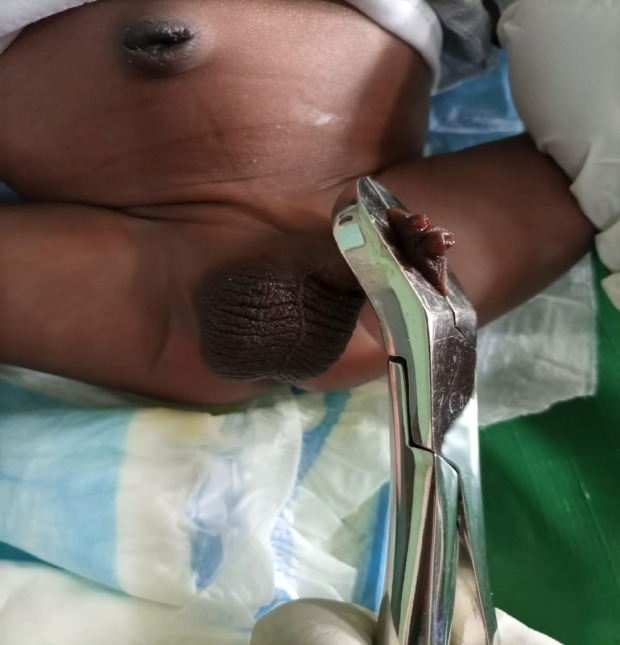
Bone-cutter circumcision

**Figure 3:**
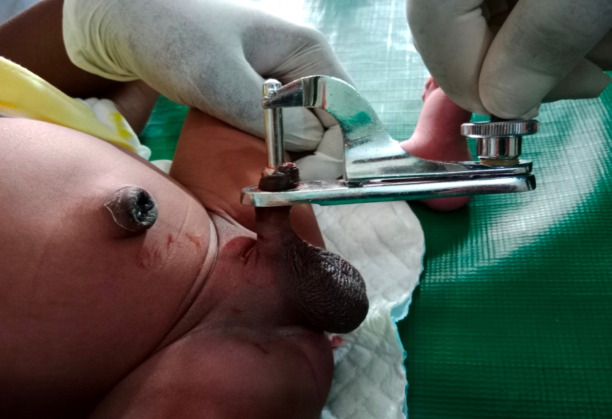
Gomco Clamp Circumcision
